# Self-care interventions and practices as essential approaches to strengthening health-care delivery

**DOI:** 10.1016/S2214-109X(22)00451-X

**Published:** 2022-10-25

**Authors:** Manjulaa Narasimhan, Mandip Aujla, Wim Van Lerberghe

**Affiliations:** a Department of Sexual and Reproductive Health and Research, WHO, 1211 Geneva, Switzerland; b Department of Health Systems Policies and Workforce, WHO, 1211 Geneva, Switzerland

Self-care gained visibility and prominence during the COVID-19 pandemic as people all over the world started to practise hand hygiene, wear a mask, and perform self-tests at home. As a cornerstone of the global response, policy makers understood the important role of individuals and communities in reducing the transmission of the virus, related mortality and morbidities, and in relieving the strain on health systems. The public had a vital role in ensuring continuity of health and social care. The pandemic also highlighted the need to give people more tools to take charge of their own health if inefficiencies across health systems and inequities in access to care are to be addressed.

There is a growing evidence base on the effectiveness of self-care interventions in the fields of communicable diseases, non-communicable diseases, mental health, and sexual and reproductive health and rights. Guidelines exist covering conditions including depression, drug and alcohol use, stress management, migraine, hypertension, coronary heart disease, and HIV, among others.^1^, ^2^, ^3^ WHO's living guideline on self-care interventions has consolidated current global policy experience and information, including on COVID-19 self-testing. The guideline provides evidence-based recommendations on how the potential of self-care interventions to improve health and social well-being can be harnessed to improve health coverage.^4^

Health workers have a key role in realising the potential of self-care interventions; their authority can be decisive for how self-confidently people care for themselves and those around them.^5^ The basic principles for health workers to promote self-care are well established: collaborative problem definition, joint targeting and goal setting, and active and sustained follow-up by skilled staff.^6^ In settings where health care functions well, personal relations of trust between health workers and their clients provide a foundation to support self-care in the community. Trust generates opportunities to improve health literacy, provide access to educational resources and financial, social, and lay support networks, and to assist navigation of complex health and social care systems. Trust facilitates support for self-management of care, such as self-administration of injectable contraceptive, self-testing for HIV or COVID-19, human papillomavirus self-sampling for cervical cancer screening, or self-monitoring of blood pressure or glycaemia.

The COVID-19 pandemic has highlighted the crucial role health professionals, particularly those working in primary care settings, can have in helping people to manage information overload, deal with misinformation, and adopt rational, healthy behaviours.

However, the capacity of health workers for supporting their clients' self-care effectively and safely is not a given. Many are poorly equipped and trained to do so.^7^ Health workers might be unaware of what options for self-care exist or the range of self-care resources and tools they could suggest.^8^ The way health workers are paid could disincentivise their work in empowering their clients because supporting self-care might require time and effort that is not accounted for in remuneration schemes. The working environment might fail to provide the necessary tools, or even discourage the people-centered approaches required for effective task-sharing; reporting systems, for example, often fail to account for efforts in support of self-care. Furthermore, self-care challenges the inherent power dynamics and gender and sociocultural biases between health workers and their clients, and might be seen as a threat to occupational status. It can be unsettling for health workers to hand over care that would otherwise be provided by, or under, their supervision, and to trust that clients can self-manage their own health.

These barriers to self-care make it all the more important for health authorities to identify what can be done to improve health workers' ability to support people in self-managing their health.^9^ Panel presents a set of policy interventions that should enable health workers to more readily understand, support, and promote self-care in their community. Most of the skills and resources needed are common to the management of a variety of health conditions.^10^ These interventions dovetail with efforts required for the transition towards integrated and people-centered care.

Panel **Ten interventions health authorities can implement to equip health workers with the skills and capacity to support self-care interventions and practices**

1 Ensure that curricula for training and continuous education of health workers gives due importance to self-care.
2 Review organisational obstacles and disincentives (eg, working conditions and resources).
3 Ensure conditions for continuity of a personal relation of trust between a client and provider.
4 Coordinate and simplify user-friendly client pathways.
5 Incorporate self-care in protocols for management of priority health conditions.
6 Include a self-care dimension in information, referral, and review systems.
7 Provide training for health workers in skills for promoting self-management and problem-solving education, group facilitation, consultation technique, adult learning, and communication.
8 Ensure staff awareness of available instruments, community resources, and social care resources.
9 Incorporate self-care support efforts in staff appraisal and remuneration systems.
10 Provide information on prevalence and trends of self-care practice in the target population.

Policy makers need to give appropriate space to self-care interventions and do so on the basis of better evidence and information, and with better instruments, than are currently available. If progress can be made, countries will be in a better position to design rational yet contextualised policies towards implementation of self-care interventions and attainment of universal health coverage, with more people-centered and close-to-home care. One of the many lessons we have learned from the COVID-19 pandemic is that self-care interventions are too important to be left to improvisation and should be built into routine health care.

We declare no competing interests.
